# Detection of heterozygous c.1708C>T and c.1978C>G thyroid peroxidase (TPO) mutations in Iraqi patients with toxic and nontoxic goiter

**DOI:** 10.1007/s00580-012-1572-9

**Published:** 2012-08-08

**Authors:** A. H. M. AL-Faisal, I. J. AL-Ramahi, I. A. Abudl-Hassan, A. T. Hamdan, S. Barusrux

**Affiliations:** 1 Genetic Engineering and Biotechnology Institute (GEBI), Baghdad, Iraq; 2 Research and Development of Medical Diagnostic Laboratories (CMDL), Al-Razi Centre for Medical Diagnostic kits Production, Ministry of Industry, Baghdad, Iraq; 3 Medical College, University of Basrah, Basrah, Iraq; 4 Faculty of Associated Medical Sciences, Khon Kaen University, Khon Kaen, 40002 Thailand

**Keywords:** Thyroid disorders, TPO, c.1708C>T, c.1978C>G, Mutation

## Abstract

Sixty-three Arabic patients (16 males and 47 females) with thyroid toxic and nontoxic goiter who attended the endocrinologist in Nuclear Medicine Hospital and Al Yarmok Nuclear Medicine Department in Baghdad, Iraq were examined for thyroid peroxidase (TPO) gene mutations. A total of ten heterozygous mutations have been identified in the human *TPO* gene associated with thyroid toxic and nontoxic goiter. These mutations involved transition or transversion of cysteine either by thymine or guanine at the position 1708 of the exon 10 (c.1708C>T) and the position 1978 of the exon 11 (c.1978C>G). From a total of ten detected mutations, two c.1978C>G mutations were detected in nontoxic goiter patients and eight (two c.1708C>T and six c.1978C>G mutations) were detected in toxic goiter. In conclusion, this study identified ten TPO mutations associated with toxic and nontoxic goiter that have not been yet reported in Iraq, and most of them are detected among females (90 %) and adults age between 30 and 50 years old (80 %).

## Introduction

Thyroid peroxidase (TPO) enzyme is a thyroid-specific glycosylated hemoprotein with a short transmembrane domain that binds it to the apical membrane of the thyrocyte (Park and Chatterjee 2005), with the catalytic part facing inside the follicle. It consists of 933 amino acids that are encoded by an mRNA of 3,048 nucleotides (Park and Chatterjee 2005). Published molecular genetic studies suggest that *TPO* gene mutations are one of the most common causes of thyroid dyshormonogenesis, with several different inactivating mutations being identified in patients with total iodide organification defects (Yardena et al. 2007). The inheritance is autosomal recessive. *TPO* gene mutations are also infrequently reported in patients with milder thyroid hormone insufficiency or partial iodide organification defects (Taurog 2000).

More than 50 TPO gene mutations have been identified including deletion, insertion, or change in DNA building blocks (Hamosh et al. 2005). Some of these mutations led to an abnormally thyroid peroxidase enzyme that breaks apart before it can be inserted into the cell membrane. Other mutations change the enzyme's three-dimensional shape, preventing it from functioning properly within the cell membrane and causing the nonaddition of the functional thyroid peroxidase iodine taken up by the thyroid gland to thyroglobulin (Avbelj et al. 2007; Yardena et al. 2007; Deladoe et al. 2008).

As a result, the production of thyroid hormones is absent or reduced, leading to the features of congenital hypothyroidism, goiter, and other thyroid disorders. The absence of TPO activity implicates the inability to iodinate tyrosine residues in TG and to couple these residues to form thyroid hormones, mainly T_4_ and some T_3_ (Taurog 2000). The most prevalent cause of inherited defects in thyroid hormone synthesis is believed to be due to TPO deficiency (Mangklabruks et al. 1991). Goiter is a thyroid gland enlargement that occurs in more than 10 % of a population (Bahn and Castro 2011). Two major types are distinguished in goiter, and both of them result from a change in thyroxin (T_3_) and triiodothyronine (T_4_) hormone levels. Nontoxic goiter is an enlargement of gland without any increased secretion of thyroid hormones but with high level of thyroid-stimulating hormone (TSH) (Hegedus and Gerber 2001). Toxic goiter occurs in three forms, namely, Graves' disease, toxic adenoma, and toxic nodular goiter, which result mainly from a hypersensitivity reaction to an autoantibody IgG that acts on surface receptors for TSH of thyroid epithelium and which are associated with high levels of thyroid hormones and with a decreased release of TSH (Chardes et al. 2002).

Many goiter patients with defects in the thyroid hormone biosynthesis have a mutation in the TPO gene (Gillam and Kopp 2001). Other genes that were also implicated in goiters include those encoding thyroglobulin (RubioIleana et al. 2009), pendrin (PDS) (Everett et al. 1997), thyroid oxidase 2 (Moreno et al. 2002), and RET (Hedayati et al. 2011).

The human TPO gene contains 17 exons and covers approximately 150 kb of chromosome 2p25 (Endo et al. 1995; Bakker et al. 2001). The first detected mutation in the TPO gene was a homozygous GGCC insertion–duplication at position 1186 in the eighth exon of the TPO gene (c.1186_1187insGGCC) (Abramowicz et al. 1992) resulting in a frameshift that generates a stop codon in exon 9, which would result in a truncated protein. Several other TPO mutations have been reported (Kotani et al. 1999; Pannain et al. 1999; Santos et al. 1999; Ambrugger et al. 2001; Kotani et al. 2003; Niu et al. 2002; Umeki et al. 2002; Wu et al. 2002). To date, more than 50 mutations in the TPO gene have been described, resulting in a variable decrease in TPO bioactivity (Hamosh et al. 2005). Most of these mutations lead to a total iodine organification defect which is the most severe and common condition leading to dyshormonogenesis of the thyroid gland. Yet no evident statistics data are available about the epidemiological profile of thyroid disease in Iraq; nevertheless, the size of problem is great. The current study aimed to detect some TPO mutations associated with goiter in Iraqi patients.

## Materials and methods

### Subjects

Sixty-three Arabic patients (16 males and 47 females) with thyroid toxic and nontoxic goiter who attended the endocrinologist in Nuclear Medicine Hospital and Al Yarmok Nuclear Medicine Department in Baghdad, Iraq as well as 25 (12 males and 13 females) Arabic healthy individuals who served as a control were selected. Healthy controls and patients' ages ranged from 17 to 79 years. The study was carried out in Baghdad, Iraq from July 2009 to October 2009.

### Inclusion and exclusion criteria

The inclusion and exclusion criteria were applied as follows:

- Inclusion criteria: Patients of any age or sex with known or suspected thyroid abnormalities and newly diagnosed and untreated patients
- Exclusion criteria: Children, pregnant or lactating women, treated patients, and patients with thyroid carcinoma, cardiac problems, and neurological disorders

### Toxic and nontoxic goiter patients and healthy control

Toxic and nontoxic goiter patients were distinguished according to the levels of thyroid hormones. The toxic and nontoxic goiters were simply diagnosed according to the level of T_3_, T_4_, and TSH hormones. Nontoxic goiter is a simple enlargement of the thyroid gland without any increased secretion of thyroid hormones (T_3_ = 1.28 ± 0.11 nmol/l, T_4_ = 87.91 ± 4.32 nmol/l, TSH = 1.61 ± 0.12 μIU/l). In contrast, toxic goiter occurs with a rise in both T_3_ and T_4_ levels (T_3_ = 1.94 ± 0.32, T_4_ = 100.3 ± 4.51) which results in reduced TSH release (0.61 ± 0.43), while healthy control was defined as person who has no known signs or symptoms of thyroid disorders and with normal levels of thyroids hormones (T_3_ = 1.44 ± 0.13 nmol/l, T_4_ = 89.76 ± 4.39 nmol/l, TSH = 1.32 ± 0.19 μIU/l) at the time of the study.

### Ethical use of data

Informed consent was obtained from all the study participants, and the guidelines set by the ethics committee of our institute and hospitals were applied.

### Statistical analysis

The data were subjected to chi-square analysis using statistical analysis system SAS (2004) program.

### Isolation of genomic DNA

Three milliliters venous blood was collected in EDTA tubes, and genomic DNA was extracted according to DNA extraction kit protocol from Promega, Canada. The DNA concentration and purity were estimated according to Sambrook et al. (1989).

### Locked nucleic acid primer PCR

Locked nucleic acid (LNA) primer PCR was carried out according to Obika et al. (1998), Singh et al. (1998), and Koshkin et al. (1998). Two TPO common mutations were selected in this study (Table 1). The mutations primers were designed and identified using NCBI tools. These mutations include 1708C>T and 1978C>G located in exons 10 and 11, of *TPO*, respectively (Bikker et al. 1997; Kotani et al. 1999). The modified LNA primers were used for the detection of *TPO* gene mutations. The design of these primer sequences was done using GeneFisher and OligoAnalyzer (https://www.idtdna.com/analyzer/Applications/ OligoAnalyzer; http://lnatools.com; http://biowww .net /protocols/index.php) and primer BLAST programs in the Faculty of Associated Medical Sciences/Kohn Kean University, (under license from Bio Basic Inc., Thailand). Degenerated primers can be designed depending on gene sequences homology. GeneFisher and OligoAnalyzer are interaction web-based programs for designed degenerated primers.

**Table 1:** LNA primer sequences and LNA base modification used for PCR amplification of *TPO* gene

Mutation	LNA primer forward (5′→3′)	LNA primer reverse (5′→3′)
*TPO* gene		
Exon 10		
1708C>T	FW-TPO gtggtttggacccactaatac	RW-TPO-23 cctgggaggttcagaacc
	FM-TPO gtggtttggacccactaatat	
Exon 11		
1978C>G	FW-TPO cctggacttgtacaagcatcc	RW-TPO-26 cgtcccattctaagtgctacg
	FM-TPO cctggacttgtacaagcatcg	

### Molecular analysis of *TPO* mutations

Targeted DNA was amplified by two LNA primer PCR reactions using modified primers: one for wild type (normal primer) and the other complimentary to the mutation to be detected (mutation primer) (Old 1996; Ye et al. 2001). The presence of product in wild type and mutant was determined as heterozygosity, the presence of only mutant band refers to homozygosity of the mutation, and the presence of the wild-type primer band only refers to normal position (no mutation) (Najmabadi et al. 2001). The optimum reaction conditions of PCR were listed in Table 2.

**Table 2:** The LNA PCR cycles for amplification conditions

Program step	Temperature (°C)	Time	No. of cycles
Preheat	95	10 min	1
Denaturation	95	30 s	30
Annealing	56	30 s	
Extension	72	30 s	
Termination	92	10 min	1
	30	3 min	

**Fig. 1:**
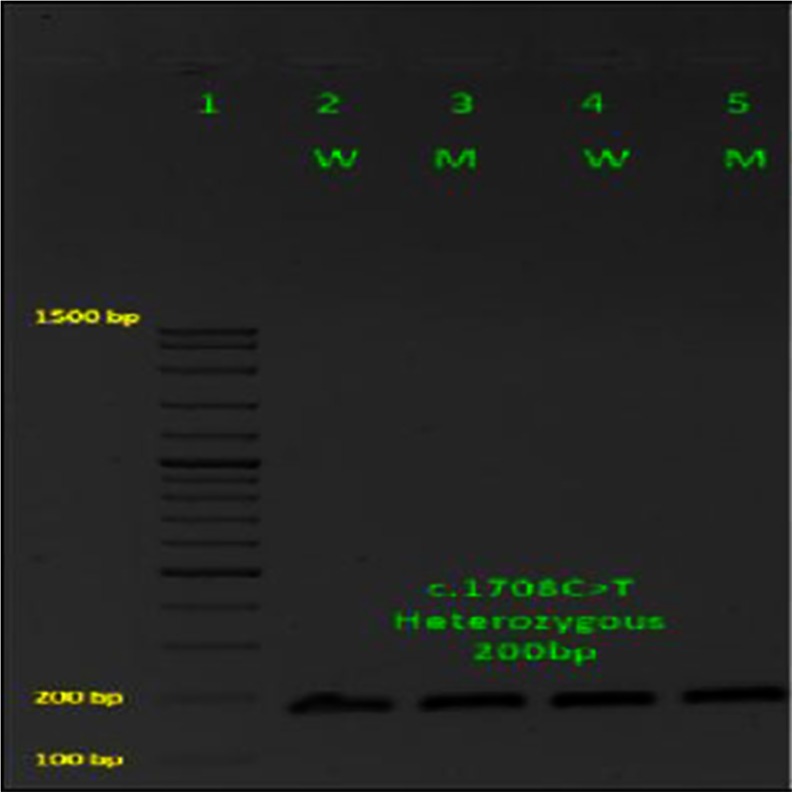
Ethidium bromide-stained 1 % agarose gel electrophoresis carried out for 45 min at 100 V. Screening for of DNA samples of patients and control for TPO gene c.1708C>T mutations by LNA primer PCR. *Lane 1* marker, *lanes 2* and *4* wild controls, *lanes 3* and *5* mutants from thyroid toxic goiter sample

The LNA primer PCR products and the ladder marker were resolved by 1 % agarose gel electrophoresis at 100 V for 45 min. The gel was stained with an ethidium bromide solution (0.5 μg/ml) and visualized on a UV transilluminator and then photographed (Fig. 1) using a GeneFlash gel documentation system (Old 1996).

## Results

The age groups in patients and healthy control samples were classified as less than 30 years, between 30 and 50 years, and more than 50 years; 27.3 % (24) of subjects were less than 30 years including 4 healthy, 7 with toxic goiter, and 13 with nontoxic goiter; 55.7 % (49) were at the age between 30 and 50 years including 16 healthy, 16 with toxic goiter, and 17 with nontoxic goiter; and 17 % (15) were at the age more than 50 years including 5 healthy, 8 with toxic goiter, and 2 with nontoxic goiter. Among the 88 subjects, 68 % (60) were females and 32 % (28) were males (Table 3).

**Table 3:** Distribution of thyroid disorders patients according to age group and sex

Groups	Age (years)				Sex	
	>30	30–50	<50	Total	Male	Female
Healthy control	4	16	5	25	12	13
Toxic goiter	7	16	8	31	12	19
Nontoxic goiter	13	17	2	32	4	28
Total	24	49	15	88	28	60
*X* ^2^	6.83**				4.21**	

**Fig. 2:**
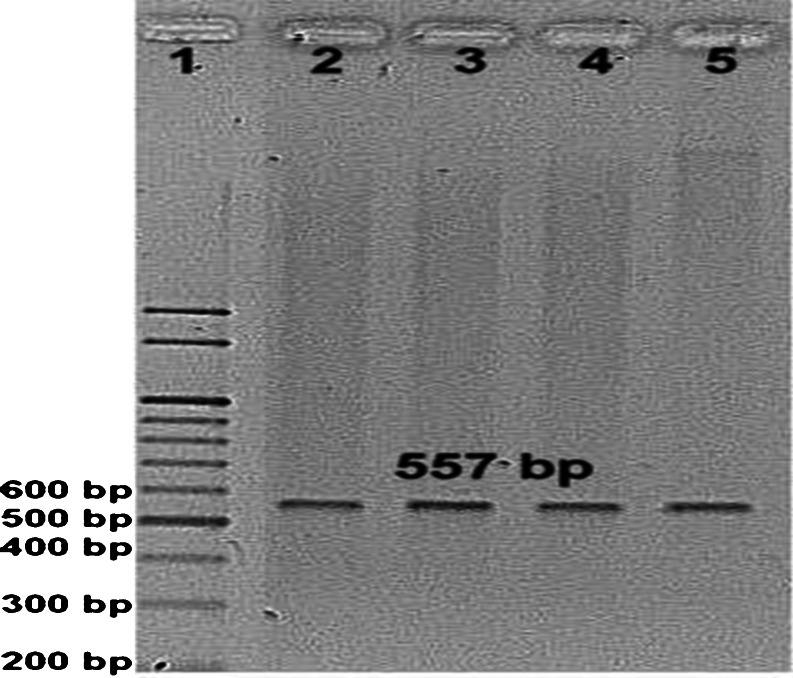
Ethidium bromide-stained 1 % agarose gel electrophoresis carried out for 45 min at 100 V. Screening of DNA samples of patients for TPO gene c.1978C>G mutations by LNA primer PCR. *Lane 1* marker, *lanes 2* and *4* wild control, *lane 3* mutant from thyroid toxic goiter samples, *lane 5* mutant from thyroid nontoxic goiter (557 bp)

No TPO mutations were detected in healthy samples. A total of ten mutations have been identified in the human *TPO* gene associated with thyroid toxic and nontoxic goiter. These mutations were involved transition or transversion of cysteine either by thymine or guanine at the position 1708 of the exon 10 (c.1708C>T) and the position 1978 of the exon 11 (c.1978C>G). From a total of ten detected mutations, two c.1978C>G mutations were detected in nontoxic goiter patients and eight (two c.1708C>T mutations and six c.1978C>G mutations) were detected in toxic goiter patients (Table 4) (Figs. 1 and 2).

**Table 4:** Distribution of TPO mutations among thyroid disorders patients

Groups	c.1708C>T	c.1978C>G	Total	No. of patients
Healthy control	0	0	0	25
Toxic goiter	2	6	8	31
Nontoxic goiter	0	2	2	32
Total	2	8	10	88

Most of the identified mutations (80 %) were seen in ages between 30 and 50 years, and the percentage of the rest of the mutations was in the patients with over 50 years old (20 %); 90 % of these mutations were distributed among females (Tables 5 and 6).

**Table 5:** Distribution of TPO mutations among thyroid disorders patients according to sex

Groups	c.1978C>G		c.1708C>T	
	Male	Female	Male	Female
Healthy control	0	0	0	0
Toxic goiter	1	5	0	2
Nontoxic goiter	0	2	0	0
Total	1	7	0	2
*X* ^2^	1.22**

**Table 6:** Distribution of TPO mutations among thyroid disorders patients according to age

Groups	c.1978C>G		c.1708C>T	
	Age (years)			
	30	30–50	>50	Total
Healthy control	0	0	0	0
Toxic goiter	0	7	1	8
Nontoxic goiter	0	1	1	2
Total	0	8	2	10
*X* ^2^	1.42**			

## Discussion

The majority of the subjects in the current study were adults whose age ranged between 30 and 50 years old (55.7 %) and the incidence was higher in females than males (68.2 %). Goiter incidence was associated with age between 30 and 50 years at diagnosis in both females and males. The frequency of goiter was higher in females than males (74.6 % (47) of 63, 25.4 % (16) of 63). These results indicate that the age and sex were related with prevalence and incidence of thyroid disorders and higher with middle ages and in women than in men. These observations are in accordance with the previous work that the majority of thyroid disorders are common in adults aged between 30 and 50 years old and the incidence was higher in females than males (Nordyke et al. 1988; Gabriel et al. 2009). Other studies were reported that prevalence of goiter has a maximum for subjects aged 10 years old (Nils et al. 2002). A more constant goiter prevalence or even the decreasing prevalence of goiter with age has been found (Brander et al. 1989; Miki et al. 1993; Brander et al. 2000; Papini et al. 2002).

The results of mutations detected in the current work revealed that the DNA instability is very high in thyroid toxic goiter (80 %) indicated that thyroid toxic goiter could be the final step to produce thyroid carcinoma. The most prevalent mutation is the c.1978C>G which represents 80 % of the total detected TPO mutations which express the importance of this mutation in thyroid disorders. Most of the TPO mutations are detected among females (90 %) and adults who aged between 30 and 50 years old (80 %). These results are in agreement with other studies which found that thyroid disorders are more common in females than males (Elahi et al. 2005; Morganti et al. 2005; Manji et al. 2006; Ahmed et al. 2009; Lamfon 2008; Jayshree and Ismail 2012).

Such DNA instability was also detected by others in toxic goiter and in thyroid cancer. Toxic goiter has been found to increase the risk to develop a thyroid lymphoproliferative disease such as thyroid lymphoma (Giusti et al. 2010) or hyperplasia (Gimm 2001). Thyroid cancer has been shown to display a high genetic instability (Alzahrani et al. 2006) which found to be correlated with the aggressiveness of the tumor and the presence of relapse in patients with thyroid cancer (Targovnik et al. 1993; Targovnik et al. 2001; Kanou et al. 2007).

The most prevalent cause of genetic abnormalities in thyroid hormone synthesis is believed to be due to TPO deficiency (Mangklabruks et al. 1991). The main cause of such deficiency is TPO exon mutations. The most frequent mutations are those occurred in exons 8, 9, 10, 11, 13, and 14 (Abramowicz et al. 1992; Medeiros-Neto et al. 1993; Bikker et al. 1994, 1995, 1997; Bakker et al. 2000, 2001; Avbelj et al. 2007; Deladoey et al. 2008).

Both c.1708C>T and c.1978C>G mutations were also detected to be associated with thyroid disorders (Bikker et al. 1997; Kotani et al. 1999). Different types of TPO mutation were identified and led to abnormal peroxidase (Hamosh et al. 2005; Avbelj et al. 2007; Magdalena et al. 2007; Yardena et al. 2007; Deladoe et al. 2008; Doga et al. 2009).

In conclusion, this study identified ten TPO mutations associated with nontoxic and toxic goiter that have not been yet reported in Iraq, and most of them are detected among females (90 %) and adults aged between 30 to 50 years old (80 %).
